# Navigating the precision gap: A critical perspective on large language models for pancreatic cyst management

**DOI:** 10.1016/j.clinsp.2026.100970

**Published:** 2026-04-24

**Authors:** Ilker Sengul, Demet Sengul

**Affiliations:** a Thyroidology Unit, Giresun University Faculty of Medicine, Turkey; b Division of Endocrine Surgery, Giresun University Faculty of Medicine, Turkey; c Department of General Surgery, Giresun University Faculty of Medicine, Turkey; d Department of Pathology, Giresun University Faculty of Medicine, Turkey

*Dear Editor,*

The management of pancreatic cystic lesions remains a complex challenge, characterized by the often-divergent recommendations of societal guidelines. This perspective evaluates the clinical and ethical implications of integrating advanced decision-support models into the surgical workflow. We submit this correspondence concerning the thoughtful and comprehensive reply^1^ to our initial commentary, entitled “Reply to: Exegesis on using a customized GPT to provide guideline-based recommendations for the management of pancreatic cystic lesions”^[^^2^ We sincerely appreciate Mr. Yuri Gorelik's insightful comments and clarifications, which further enrich the ongoing discourse on the application of Large Language Models (LLMs) in clinical practice^[^^1^ We concur with the assertion that their initial work^3^ was a critical pilot study aimed at evaluating the potential of LLMs to navigate complex clinical scenarios characterized by conflicting guidelines and practice variability. Their proof-of-concept study demonstrated that a custom GPT agent could integrate diverse data and provide guideline-based recommendations for managing pancreatic cysts, yielding results as comparable to those of expert opinions. This initial work highlights the promising utility of advanced LLMs in enhancing clinical decision-making. We are particularly encouraged to learn that larger-scale studies using real-world data are currently underway, directly addressing the critical area of sample size and clinical validation. The transition from simulated scenarios to actual clinical cases is a vital step forward, necessary for validating the utility of custom GPTs in a real-world setting.

## Critical analysis and implementation challenges

While the technological advancement is evident, a crucial aspect that requires deeper scholarly engagement is the rigorous validation and safe integration of these models. We advocate for a staged validation pathway: from simulation to prospective multi-center trials. Furthermore, accountability must be grounded in frameworks like the EU AI Act to address the current ambiguity in clinical liability. Comparability must be established through robust metrics, including statistical power and transparent definitions of “expert” consensus and inter-rater reliability, which remain critical variables in validation frameworks. Furthermore, leveraging the advanced reasoning capabilities of newer LLMs (reasoning models) is profoundly significant. The ability to review and study the model's reasoning process enhances transparency and addresses the inherent “black box” problem. However, this transparency is only a partial solution when facing the critical challenge of model safety. The potential for model hallucination ‒ generating factually incorrect or clinically misleading information ‒ poses an unacceptable risk in diagnostic and management decision support. Before deployment, the field requires dedicated frameworks to test these models against corner cases and contradictory data sets to ensure they are generalizable beyond the specific guidelines on which they were trained.

A rigorous discussion of clinical implementation and safety must balance the enthusiastic adoption of AI:

1. Regulatory Pathways: The absence of clear regulatory pathways (e.g., FDA or CE marking equivalent) for LLM-based clinical decision support tools means that accountability for model performance is undefined.

2. Liability and Accountability: A fundamental challenge remains concerning liability: who is responsible if an AI-generated recommendation leads to a suboptimal or poor patient outcome? This uncertainty must be resolved through clear legal and ethical guidelines before widespread integration into clinical workflows.

3. Data Privacy and Security: Utilizing real-world patient data for ongoing model refinement necessitates adherence to stringent global data privacy standards (e.g., HIPAA, GDPR).

4. Clinical Integration: Practical hurdles include user interface design, computational resource requirements, and ensuring seamless adoption into diverse clinical settings.

## Conclusion and future directions

The future of pancreatology necessitates a synergy between human expertise and digital precision. Establishing robust legal and ethical safeguards is the next critical step for research and policy. Given the well-documented inconsistencies among societal guidelines and the wide variation in surveillance and management practices for pancreatic cysts, an integrated LLM-based solution holds promising potential in this crucial clinical field [1, 2, 3] This issue merits continued, critical investigation supported by prospective, multi-center trials. We thank Yuri Gorelik and colleagues for advancing this critical area of research. We look forward to the findings from future studies, which we believe will further demonstrate the promising influence of LLMs[4] on patient management and clinical decision-making.

## Abbreviations

GPT, Generative Pre-trained Transformer; LLM, Large Language Model; AI, Artificial Intelligence.

## Data availability

The datasets used and/or analyzed during this study are available from the corresponding author upon reasonable request.

## Ethics approval

Not applicable.

## Consent to participate

Not applicable.

## Consent to publish

Not applicable.

## Code availability

Not applicable.

## Authors’ contributions

Ilker Sengul: Conceptualization; formal analysis; writing-original draft; writing-review & editing.

Demet Sengul: Conceptualization; formal analysis; supervision; writing-original draft; writing-review & editing.

## Funding

None declared.

ARTICLE INFO

*Article history*:

Received 16 October 2025

Accepted 25 January 2026

## Declaration of competing interest

The authors declare no conflicts of interest.
